# Who Is Joseph Lobosco?

**Published:** 1921-07

**Authors:** Wm. Ehlers

**Affiliations:** Atlantic Rubber Mfg. Corporation, 239 Fourth Ave., New York City.


### WHO IS JOSEPH LOBOSCO?

Does he want $39.24? We have an entry in our books under date of December 30, 1918, in favor of one Joseph Lobosco $39.24. This amount has been received, and unfortunately our bookkeeper neglected to enter the place from which the money came. At the time there was no record of an order, and it was decided to wait for the order, which we expected to come, as a matter of course. While waiting for this order the item was forgotten. It now turns up in our books, and troubles our conscience.

If Joseph Lobosco is still alive, or if anybody knows who he is, will he kindly step forward, and communicate with the undersigned, so that he can quiet his conscience?

It is presumed that the money came by postal order from a foreign country, but without data it is impossible to locate the source of the remittance.

Atlantic Rubber Mfg. Corporation,
(Successors to Traun Rubber Co.,)
Wm. Ehlers, Sec.

239 Fourth Ave., New York City.

